# Rapidly progressive amyotrophic lateral sclerosis is associated with microglial reactivity and small heat shock protein expression in reactive astrocytes

**DOI:** 10.1111/nan.12525

**Published:** 2018-11-23

**Authors:** R. P. Gorter, J. Stephenson, E. Nutma, J. Anink, J. C. de Jonge, W. Baron, M.‐C. Jahreiβ, J. A. M. Belien, J. M. van Noort, C. Mijnsbergen, E. Aronica, S. Amor

**Affiliations:** ^1^ Department of Pathology, Amsterdam Neuroscience Amsterdam UMC VU University Medical Center Amsterdam The Netherlands; ^2^ Centre for Neuroscience and Trauma Blizard Institute Barts and the London School of Medicine and Dentistry Queen Mary University of London London UK; ^3^ Department of (Neuro)Pathology, Amsterdam Neuroscience Amsterdam UMC University of Amsterdam Amsterdam The Netherlands; ^4^ Section Molecular Neurobiology Department of Biomedical Sciences of Cells & Systems University Medical Center Groningen University of Groningen Groningen The Netherlands; ^5^ Deltacrystallon Leiden The Netherlands

**Keywords:** amyotrophic lateral sclerosis, astrocytes, HSPB, inflammation, small heat shock proteins, TDP‐43 pathology

## Abstract

**Aims:**

Amyotrophic lateral sclerosis (ALS) is a chronic neurodegenerative disease characterized by progressive loss of motor neurons, muscle weakness, spasticity, paralysis and death usually within 2–5 years of onset. Neuroinflammation is a hallmark of ALS pathology characterized by activation of glial cells, which respond by upregulating small heat shock proteins (HSPBs), but the exact underlying pathological mechanisms are still largely unknown. Here, we investigated the association between ALS disease duration, lower motor neuron loss, TARDNA‐binding protein 43 (TDP‐43) pathology, neuroinflammation and HSPB expression.

**Methods:**

With immunohistochemistry, we examined HSPB1, HSPB5, HSPB6, HSPB8 and HSP16.2 expression in cervical, thoracic and sacral spinal cord regions in 12 ALS cases, seven with short disease duration (SDD), five with moderate disease duration (MDD), and ten age‐matched controls. Expression was quantified using ImageJ to examine HSP expression, motor neuron numbers, microglial and astrocyte density and phosphorylated TDP‐43 (pTDP‐43+) inclusions.

**Results:**

SDD was associated with elevated HSPB5 and 8 expression in lateral tract astrocytes, while HSP16.2 expression was increased in astrocytes in MDD cases. SDD cases had higher numbers of motor neurons and microglial activation than MDD cases, but similar levels of motor neurons with pTDP‐43+ inclusions.

**Conclusions:**

Increased expression of several HSPBs in lateral column astrocytes suggests that astrocytes play a role in the pathogenesis of ALS. SDD is associated with increased microgliosis, HSPB5 and 8 expression in astrocytes, and only minor changes in motor neuron loss. This suggests that the interaction between motor neurons, microglia and astrocytes determines neuronal fate and functional decline in ALS.

## Introduction

Amyotrophic lateral sclerosis (ALS) is a progressive neurological disorder in which motor neurons in the motor cortex, brainstem and spinal cord degenerate, leading to spasticity, muscle weakness and atrophy 1. The onset of ALS is primarily diagnosed between 55 and 75 years of age 2 and characterized by focal motor deficits that rapidly accumulate, often resulting in paralysis and death due to respiratory failure within 2–5 years 3.

The majority of ALS cases are sporadic, although genetic mutations are associated with familial forms of the disease in 5–10% of cases 1. Virtually all these mutations affect genes involved in protein homoeostasis (proteostasis) 4. Although the exact cause of ALS is unknown, defects in proteostasis may play an important role in disease pathogenesis leading to the formation of toxic aggregates 5.

In response to increased or dysfunctional proteostasis, cells upregulate heat shock proteins (HSPs) to protect against cellular damage that accumulates during pathological conditions 6. HSPs are also upregulated after cellular stress, including heat, hypoxia, inflammation and oxidative stress 7. Given the strong association between protein aggregation, glial activation and neurodegeneration, the expression and role of HSPs in the central nervous system (CNS) is attracting attention in many neurodegenerative disorders 8, especially as studies show that HSPs can potentially act as neuroprotective agents 9. HSPs are classified into several families, including the large HSPA (HSP70) and HSPD1 (HSP60) and the small heat shock proteins (HSPBs) 10. Several HSPs are constitutively expressed in the CNS while others are only observed in pathological conditions such as in Alzheimer's disease, Parkinson's disease, Alexander's disease, multiple sclerosis and X‐linked adrenoleucodystrophy 11, 12, 13, 14, 15.

HSPBs are a family of 11 highly conserved proteins that act as molecular chaperones and cytoskeleton stabilizers, exerting protective effects by reducing protein misfolding and promoting degradation of misfolded proteins 16. The role of HSPBs in neurodegenerative disorders is supported by studies showing that mutations in HSPB1, 3, 5 and 8 are associated with distal hereditary motor neuropathies and myofibrillar myopathies characterized by protein inclusions 17. *In vitro*, HSPB1, 5 and 8 prevent superoxide dismutase (SOD1) and TARDNA‐binding protein 43 (TDP‐43) aggregation by promoting degradation and solubility 18, 19. *In vivo*, experimental induction of an HSPB5/8 homologue partially rescues TDP‐43 aggregation and increases life span in drosophila 20. In support of a protective role of HSPB in animal models of ALS, slow‐progressing SOD1^G93A^ mice have fivefold higher expression of HSPB5 when compared to fast‐progressing SOD1^G93A^ mice 21.

Apart from their roles as molecular chaperones, small HSPs also inhibit apoptosis and play key roles in immune regulatory pathways 22, 23. For example, HSPB5 induces an anti‐inflammatory phenotype in microglia and macrophages *in vitro* that switches to a pro‐inflammatory response in the presence of IFN‐γ 24.

Initial results of a recent clinical trial suggest that arimoclomol, an inducer of the heat shock response, might slow progression and respiratory decline in ALS patients 4. While many studies hint at the protective effects of HSPBs in neuroinflammatory diseases including ALS, little is known about HSPB expression, and how such expression is associated with motor neuron damage and glial activation in ALS.

In this study, we compared HSPB1, 5, 6, 8 and orphan small heat shock protein HSP16.2 25 expression in spinal cords of non‐neurological controls and ALS patients with short disease duration (SDD) and moderate disease duration (MDD). We show that HSPB expression by motor neurons and astrocytes is altered in ALS and that HSPB expression is associated with ALS disease duration.

## Materials and methods

### Spinal cord samples

Formalin‐fixed, paraffin‐embedded spinal cord tissue from 12 ALS cases (mean age = 65.8 years), 7 SDD (<18 months survival; mean survival = 11.1 ± 3.4 months) and 5 MDD (>24 months survival; mean survival = 62.8 ± 33.8 months) and 10 non‐neurological controls (mean age = 64.9 years) was collected at *post mortem* in the pathology department of the Academic Medical Center (University of Amsterdam), with the approval of the AMC Medical Ethical Committee and according to local legal and ethical regulations. Patients or relatives gave informed consent for autopsy and use of spinal cord tissue for research purposes. Tissue samples from cervical levels (1 sample per case), thoracic (two samples per case – except for cases 5 and 18 where one sample was available) and lumbar (one sample per case) were examined. Patient characteristics are listed in Table 1. Genetic analysis for 235 common mutations implicated in ALS revealed one case with a valosin‐containing protein mutation (case 8); and a *C9ORF72* repeat expansion was detected in one case (case 12).

**Table 1:** Patient characteristics

	Sex	Age	ALS form or NNC details	Primary onset	ALS DD (months)	Cause of death	PMD (h)
ALS							
1	F	70	sALS	Leg	6 (SDD)	Respiratory failure	3:05
2	M	63	sALS	Leg	7 (SDD)	Respiratory failure	<12
3	F	61	sALS	Arm	12 (SDD)	Euthanasia	<12
4	M	60	sALS	Arm	12 (SDD)	Euthanasia	<12
5	M	81	sALS	Respiratory	12 (SDD)	Respiratory failure	<12
6	F	84	sALS	Bulbar	13 (SDD)	Euthanasia	<12
7	F	56	sALS	Leg	16 (SDD)	Euthanasia	<12
8	F	61	sALS *(VCP mutation)*	Arm	27 (MDD)	Euthanasia	<12
9	M	43	sALS	Arm	36 (MDD)	Unknown	<12
10	F	64	fALS	Leg	57 (MDD)	Pneumonia	<12
11	M	68	sALS	Arm	87 (MDD)	Euthanasia	<12
12	M	79	sALS *(C9orf72 repeat expansion)*	Unknown	107 (MDD)	Pneumonia	<12
Controls							
13	M	60	BRICKER bladder	n.a.	n.a.	Lung embolism	<24
14	M	63	Renal carcinoma	n.a.	n.a.	Lung embolism	<24
15	F	81	Cardiac ischemia	n.a.	n.a.	Endocarditis	<24
16	F	63	Adenocarcinoma	n.a.	n.a.	Paralytic ileus	<24
17	M	69	Oesophageal carcinoma	n.a.	n.a.	Multi organ failure	<24
18	F	78	Cholangiocarcinoma	n.a.	n.a.	Multi organ failure	<48
19	M	75	COPD, pneumonia	n.a.	n.a.	Respiratory failure	<24
20	F	59	Pleuritis carcinomatosa	n.a.	n.a.	Respiratory failure	<24
21	F	47	Pancreatic carcinoma	n.a.	n.a.	Abdominal bleeding	<12
22	F	54	Carcinoma of the gallbladder	n.a.	n.a.	Acute heart failure	<24

COPD, chronic obstructive pulmonary disease; ALS, amyotrophic lateral sclerosis; fALS, familial ALS; n.a., not applicable; NNC, non‐neurological controls; PMD, *post mortem* delay; MDD, moderate disease duration; sALS, sporadic ALS; SDD, short disease duration; VCP, valosin‐containing protein.

### Immunohistochemistry

Paraffin sections were deparaffinized with xylene, rehydrated in graded ethanol solutions and washed in water. Endogenous peroxidase was blocked by incubating the slides in phosphate‐buffered saline (PBS) containing 0.3% (v/v) hydrogen peroxide for 30 min. After washing in PBS, heat‐mediated antigen retrieval was performed using either 0.01 M citrate buffer (pH 6) or ethylene diamine tetra‐acetic acid buffer (pH 9). After cooling and washing in PBS, slides were incubated for 1 h or overnight with primary antibodies (Table S1) directed to phosphorylated TDP‐43 (pTDP‐43), human leucocyte antigen receptor D related (HLA‐DR), glial fibrillary acid protein (GFAP), aldehyde dehydrogenase 1 (ALDH1), vimentin, HSPB1, HSPB5, HSPB6, HSPB8 and HSP16.2 (Table S1) diluted in normal antibody diluent (Immunologic), at room temperature. Sections were washed and incubated with the secondary antibody (Table S1): goat‐anti‐mouse horseradish peroxidase (HRP) Envision (Dako, Glostrup, Denmark) for HSPB5 and goat‐anti‐rabbit HRP Envision (Dako) for other HSPBs. After washing with PBS, the staining was developed with 3,3′‐diaminobenzidine (DAB; Dako) at a 1:50 concentration for 10 min. Slides were washed in tap water, the nuclei counterstained with haematoxylin and sections dehydrated in ascending alcohol concentrations and xylene, and mounted with Quick‐D (Klinipath, Olen, Belgium). Prior to the study, all the antibodies and relevant isotype controls 14 were used to check background and cross reactivity. Isotype controls did not reveal background staining.

### pTDP‐43 and cresyl‐violet staining

To identify motor neurons expressing pTDP‐43 aggregates spinal cord sections were stained with an antibody directed to pTDP‐43 (Table S1) as described above. After development with DAB, sections were incubated overnight with 1% cresyl‐violet solution, washed and dehydrated in ascending alcohol concentrations and xylene, then mounted with Quick‐D (Klinipath).

### Double labelling

To identify cells expressing HSPBs, double labelling was performed for HLA‐DR (microglia), oligodendrocyte transcription factor 2 (olig2) (oligodendrocytes) or vimentin (astrocytes). Sections were stained with HSPs as described above and after developing with DAB, the slides were washed and incubated with the second primary antibody for 1 h or overnight (Table S1). When primary antibodies were from the same species, the antibody directed to the first antigen of choice was detached by heating in citrate buffer for 15 min. After washing, the appropriate secondary antibody, goat‐anti‐mouse alkaline phosphatase (AP) or goat‐anti‐rabbit AP (Table S1), was applied for 1 h. After washing twice with PBS and once with tris‐buffered saline, slides were developed with liquid permanent red (LPR; Dako; 1:100) for 10 min. Slides were washed in tap water and nuclei were counterstained with haematoxylin, washed in water and mounted with Aquatex (Merck Millipore, Darmstadt, Germany). Following omission of primary antibodies, no staining was observed.

### Quantitative and statistical analysis

#### Motor neuron and pTDP‐43 pathology quantification

Spinal cord sections, immunostained for pTDP‐43 and Harris’ haematoxylin, were digitalized using the Mirax slide scanner system equipped with a 20× objective with a numerical aperture of 0.75 (3DHISTECH, Budapest, Hungary) and Sony DFW‐X710 Fire Wire 1/3″ type progressive SCAN IT CCD (pixel size: 4.65 × 4.65 μm^2^). The scan resolution of all images at 20× was 0.23 μm. After scanning, representative areas were annotated manually by a blinded observer (RPG) using pannoramic viewer software (3DHISTECH) and subsequently exported in the TIFF image format. To determine the numbers of motor neurons and motor neurons containing TDP‐43 pathology, a second blinded observer (JS) manually counted the defined areas using ImageJ (Fiji; {HYPERLINK ‘https://fiji.sc’}) 26.

#### HLA‐DR, GFAP, ALDH1, vimentin and HSP expression quantification

The extent of HLA‐DR, GFAP, ALDH1, vimentin and HSP expression in the lateral tracts and ventral horns was assessed using ImageJ. Three pictures of the left and right ventral horns and lateral corticospinal tracts at each spinal cord level (cervical, thoracic [2×], lumbar) were taken by a blinded observer (RPG) at 400× magnification using an Olympus BX41 microscope equipped with a Leica MC170 HD camera (Leica Microsystems, Heidelberg, Germany). Pictures were analysed using imagej software to calculate the percentage of DAB+ pixels per image. Arbitrary thresholds were set to eliminate background expression. Next, brown DAB staining was separated from blue haematoxylin staining and the percentage of DAB+ area was determined using the macro supplied (Appendix S1). Mean values were calculated from three images per sample. Vimentin and ALDH1 expression was mostly seen in astrocytes, but also to a lesser extent in cells that morphologically resembled foamy macrophages. To only quantify astrocyte reactivity, ImageJ was used to manually remove these cells based on morphology prior to quantification.

#### Statistical analysis

Using graphpad prism 6 (GraphPad Software, La Jolla, CA, USA; https://www.graphpad.com/scientific-software/prism/), motor neuron numbers, ventral horn surface area, absolute numbers of TDP‐43+ motor neurons, fraction of motor neurons which are TDP‐43+ as well as HLA‐DR, GFAP, ALDH1, vimentin and HSP expression in the lateral tracts and ventral horns was examined. The expression levels of the separate spinal cord levels (one cervical, one to two thoracic, one lumbar) were averaged resulting in mean values for each case. Data were checked for normal distribution and parametric or non‐parametric tests were used accordingly. Differences between ALS patients and controls were evaluated using Student's t‐test or Mann‐Whitney *U*‐test. Differences between the subgroups with SDD and MDD and controls were determined using analysis of variance or Kruskal–Wallis *H* test. When *P* < 0.05 post hoc analysis was performed using Tukey's test or Dunn's multiple comparisons test.

## Results

### Short ALS disease duration is associated with pTDP‐43 pathology and activated microglia but not motor neuron loss

**Figure 1:**
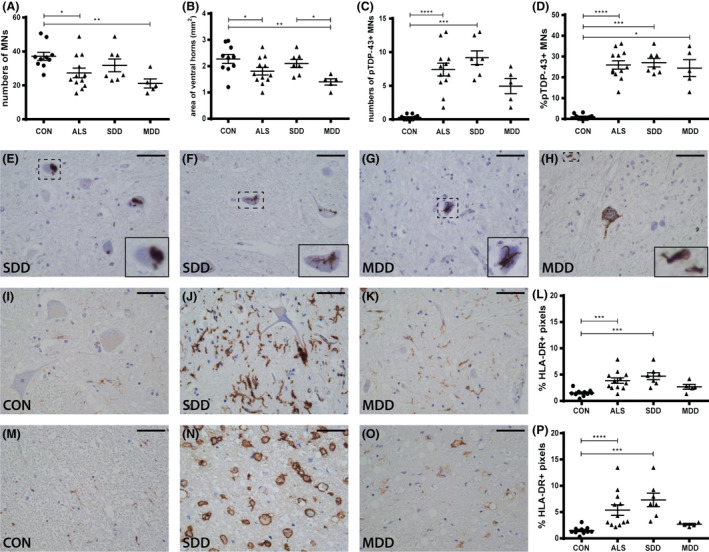
Motor neurons, pTDP‐43 inclusions and microglial activation in ALS spinal cord. Quantification of (**A**) motor neuron counts, (**B**) ventral horn surface, (**C**) number of pTDP‐43+ inclusions and (**D**) percentage of pTDP‐43+ inclusion containing motor neurons in controls and ALS patients (subgroups: SDD and MDD). pTDP‐43+ motor neurons in ALS patients with (**E**,**F**) SDD and (**G**,**H**) MDD. HLA‐DR expression in (**I**–**K**) ventral horns and (**M**–**O**) lateral tracts of controls and SDD and MDD patients. Quantification of HLA‐DR+ pixels in (**L**) ventral horns and (**P**) lateral tracts of controls and ALS patients (subgroups: SDD and MDD). Data points represent the mean value for each patient. Data are shown as mean ± SEM. Significance was analysed between ALS patients (*n* = 12) and controls (*n* = 10) with Student's *t*‐test or Mann–Whitney *U‐*test. ALS patients SDD (*n* = 7) and MDD (*n* = 5) were compared to controls (*n* = 10) using anova and Tukey's post‐test or Kruskal–Wallis *H* test and Dunn's *post hoc* multiple comparisons test. Significant data are presented (****P = <0.0001, ***P = <0.001, **<0.01, *P = <0.05). Scale bar in all pictures = 50 μm. Inserts are digitally enlarged. SDD, short disease duration; MDD, moderate disease duration; ALS, amyotrophic lateral sclerosis; HLA‐DR, human leucocyte antigen‐D related; TDP, TARDNA‐binding protein; pTDP‐43, phosphorylated TDP.

Spinal cords of ALS patients (SDD and MDD) and non‐neurological controls were characterized based on motor neuron counts (Figure 1 **A**), ventral horn surface area (Figure 1 **B**), TDP‐43 pathology (Figure 1 **C**,**D**) and HLA‐DR reactivity (Figure 1 **L**,**P**). Compared to non‐neurological controls, ALS spinal cords contained fewer motor neurons (−26.4%; *P* = 0.0172; Figure 1 **A**), but more pTDP‐43 pathology (+2406%; *P* < 0.0001; Figure 1 **C**,**D**). In patients with SDD, ventral horn motor neuron numbers were not significantly lower (−14.4%; *P* = 0.1906; Figure 1 **A**), and a significant number of neurons were pTDP‐43+ (+3001%; *P* = 0.0001; Figure 1 **C**). In the pTDP‐43+ motor neurons, the inclusions were observed as round balls or smaller string‐like aggregates (Figure 1 **E**,**F**). Motor neuron numbers (−43.1%; *P* = 0.0052; Figure 1 **A**) and ventral horn surface area (−38.4%; *P* = 0.0067; Figure 1 **B**) were significantly decreased in patients with MDD. Ventral horn surface area was significantly lower in MDD compared to SDD (*P* = 0.0314; Figure 1 **B**). The few remaining motor neurons in MDD cases appeared dystrophic and often contained extensive pTDP‐43 inclusions (Figure 1 **G**,**H**). In both SDD and MDD cases, approximately 25% of motor neurons were pTDP‐43+ (Figure 1 **D**).

HLA‐DR expression as a marker of microglia and macrophage activity (Figure 1 **L**–**P**) revealed significantly higher expression in the ventral horns (+212%; *P* < 0.0001; Figure 1 **L**) and lateral tracts (+392%; *P* = 0.0009; Figure 1 **P**) in SDD cases, but not in MDD cases. Sparse HLA‐DR+ cells were present and the surrounding tissue appeared fibrotic (Figure 1 **O**). HLA‐DR+ foamy cells were only observed in white matter and predominantly in patients with SDD (Figure 1 **N**).

### Increased astrocyte reactivity in ventral horns in ALS

**Figure 2:**
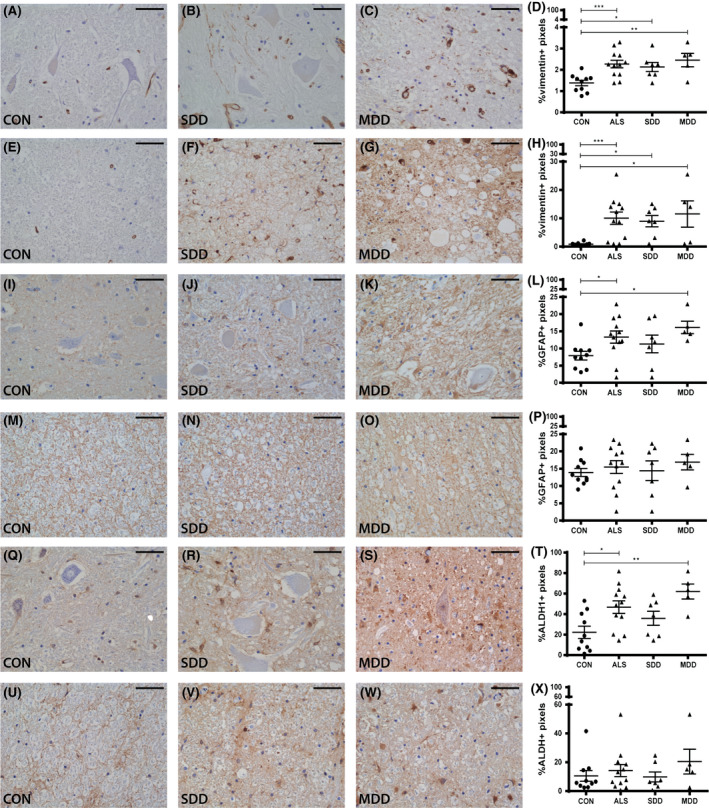
Astrocyte reactivity in ALS spinal cord. Expression (**A**–**C**) and quantification (**D**) of vimentin+ pixels in ventral horns of controls and ALS patients (subgroups: SDD and MDD). Expression (**E**–**G**) and quantification (**H**) of vimentin+ pixels in lateral columns of controls and ALS patients (subgroups: SDD and MDD). Expression (**I**–**K**) and quantification (**L**) of GFAP+ pixels in ventral horns of controls and ALS patients (subgroups: SDD and MDD). Expression (**M**–**O**) and quantification (**P**) of GFAP+ pixels in lateral columns of controls and ALS patients (subgroups: SDD and MDD). Expression (**Q**–**S**) and quantification (**T**) of ALDH1+ pixels in ventral horns of controls and ALS patients (subgroups: SDD and MDD). Expression (**U**–**W**) and quantification (**X**) of ALDH1+ pixels in lateral columns of controls and ALS patients (subgroups: SDD and MDD). Data points represent the mean value for each patient. Data are shown as mean ± SEM. Significance was analysed between ALS patients (*n* = 12) and controls (*n* = 10) with Student's *t*‐test or Mann–Whitney *U‐*test. ALS patients with short disease duration (SDD; *n* = 7) and moderate disease duration (MDD; *n* = 5) were compared to controls (*n* = 10) using anova and Tukey's post‐test or Kruskal–Wallis *H* test and Dunn's *post hoc* multiple comparisons test. Significant data are presented (****P = <0.0001, ***P = <0.001, **<0.01, *P = <0.05). Scale bar in all pictures = 50 μm. SDD, short disease duration; MDD, moderate disease duration; ALS, amyotrophic lateral sclerosis; GFAP, glial fibrillary acid protein; ALDH1, aldehyde dehydrogenase 1.

To assess astrocyte reactivity, the expression of astrocyte markers vimentin, GFAP and ALDH1 was compared between ALS cases (SDD and MDD) and controls (Figure 2). The expression of vimentin was significantly increased in ALS cases in the ventral horns (Figure 2 **A**–**D**; ALS *P* = 0.0009; SDD *P* = 0.0251; MDD *P* = 0.0042) and lateral columns (Figure 2 **E**–**H**; ALS *P* = 0.0003; SDD *P* = 0.0144; MDD *P* = 0.0177) compared to controls. An increased density of GFAP in the ventral horns in ALS cases (*P* = 0.0272) and specifically in those cases with moderate (*P* = 0.0218) but not SDD (*P* = 0.2648) was observed (Figure 2 **I**–**L**), but no differences in expression were found in the lateral tracts (Figure 2 **M**–**P**). Similarly, the density of ALDH1 was also increased in ALS cases (*P* = 0.0105) and in cases with MDD (*P* = 0.0021) but not SDD (*P* = 0.3026) in the ventral horns (Figure 2 **Q**–**T**), but not in the lateral columns (Figure 2 **U**–**X**).

### HSPB1 expression is lower in ventral horns of ALS spinal cord

**Figure 3:**
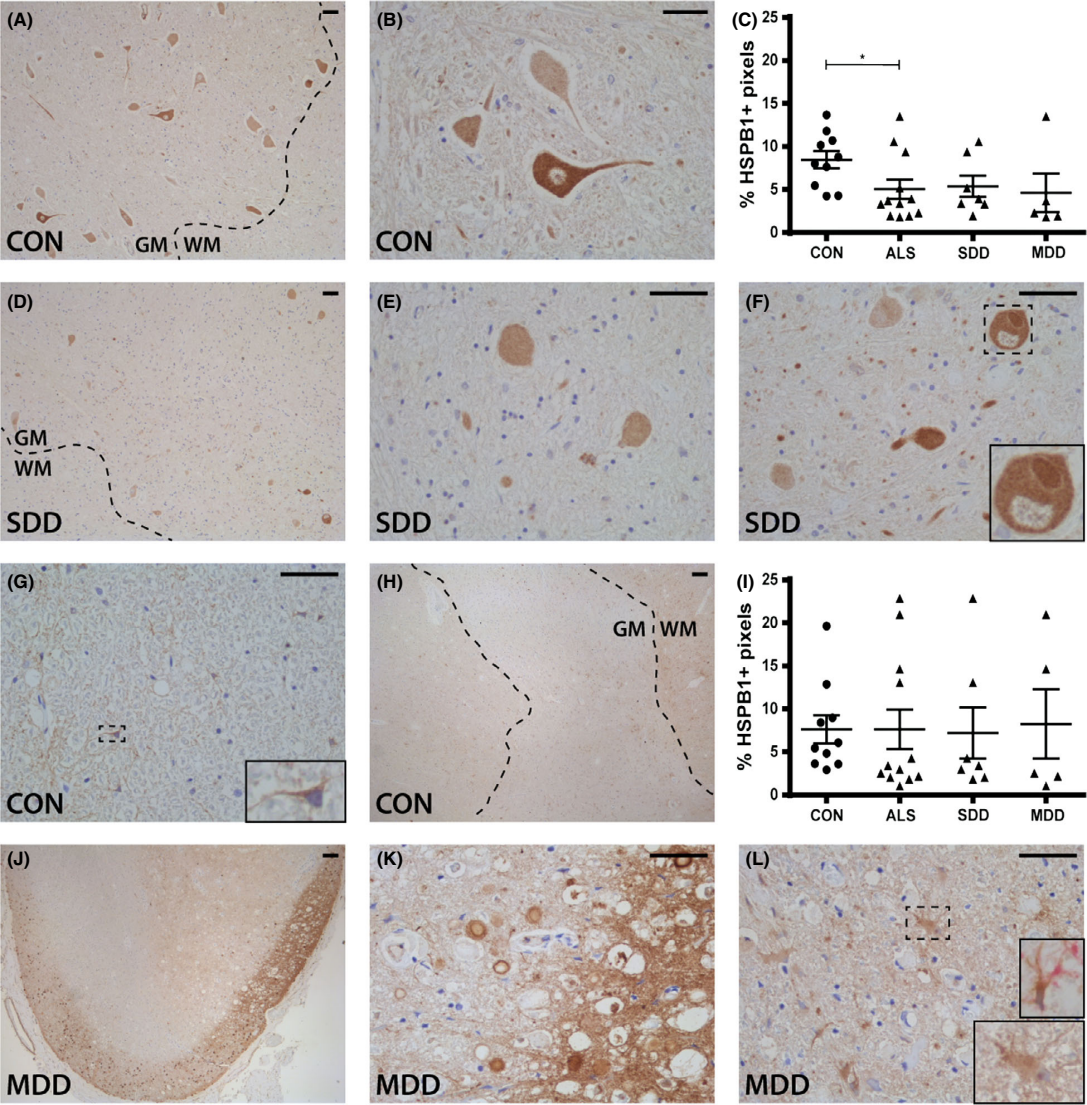
HSPB1 expression in ALS spinal cord. HSPB1 expression in (**A**,**B**,**D**–**F**) ventral horns and (**G**,**H**,**J**–**L**) lateral tracts of controls and ALS patients (subgroups: SDD and MDD) with (**L**) an insert of a vimentin+ (pink) and HSPB1+ (brown) astrocyte. Grey matter is delineated with a dotted line. Quantification of HSPB1+ pixels in (**C**) ventral horns and (**I**) lateral columns of controls and ALS patients (subgroups: SDD and MDD). Data points represent the mean value for each patient. Data are shown as mean ± SEM. Significance was analysed between ALS patients (*n* = 12) and controls (*n* = 10) with Student's *t*‐test or Mann–Whitney *U‐*test. ALS patients with SDD (*n* = 7) and MDD (*n* = 5) were compared to controls (*n* = 10) using anova and Tukey's post‐test or Kruskal–Wallis *H* test and Dunn's *post hoc* multiple comparisons test. Significant data are presented (****P = <0.0001, ***P = <0.001, **<0.01, *P = <0.05). Scale bar in all pictures = 50 μm. Inserts are digitally enlarged. SDD, short disease duration; MDD, moderate disease duration; ALS, amyotrophic lateral sclerosis; HSPB, heat shock protein B.

In controls, HSPB1 was present in cell bodies, axons and processes, but not in nuclei of motor neurons throughout the ventral and dorsal horns (Figure 3 **A**,**B**). In accordance with the motor neuron loss in ALS, fewer HSPB1+ motor neurons were observed in the ventral horns (data not shown) contributing to the decreased HSPB1 expression in ALS (*P* = 0.0384; Figure 3 **C**) although differences could not be attributed to disease duration that is SDD (*P* = 0.2261) or MDD (*P* = 0.1640). In one SDD case, HSPB1+ inclusions were seen in the neuronal bodies (Figure 3 **F**).

In control spinal cord, cells with an astrocyte‐like morphology constitutively expressed HSPB1 throughout the white matter (Figure 3 **G**) and grey matter in close proximity to the central canal (Figure 3 **H**). In most ALS patients, HSPB1 expression was similar to controls and colocalized with vimentin, but not olig2. Overall, HSPB1 expression in the lateral tracts of ALS spinal cord was similar to controls (Figure 3 **I**), however, in a small subset of ALS patients, many HSPB1+ astrocytes were observed (Figure 2 **J**) and occasionally HSPB1 was highly expressed in the subpial region (Figure 3 **K**,**L**).

### ncreased HSPB5 and HSPB8 expression in astrocytes in ALS cases with SDD

**Figure 4:**
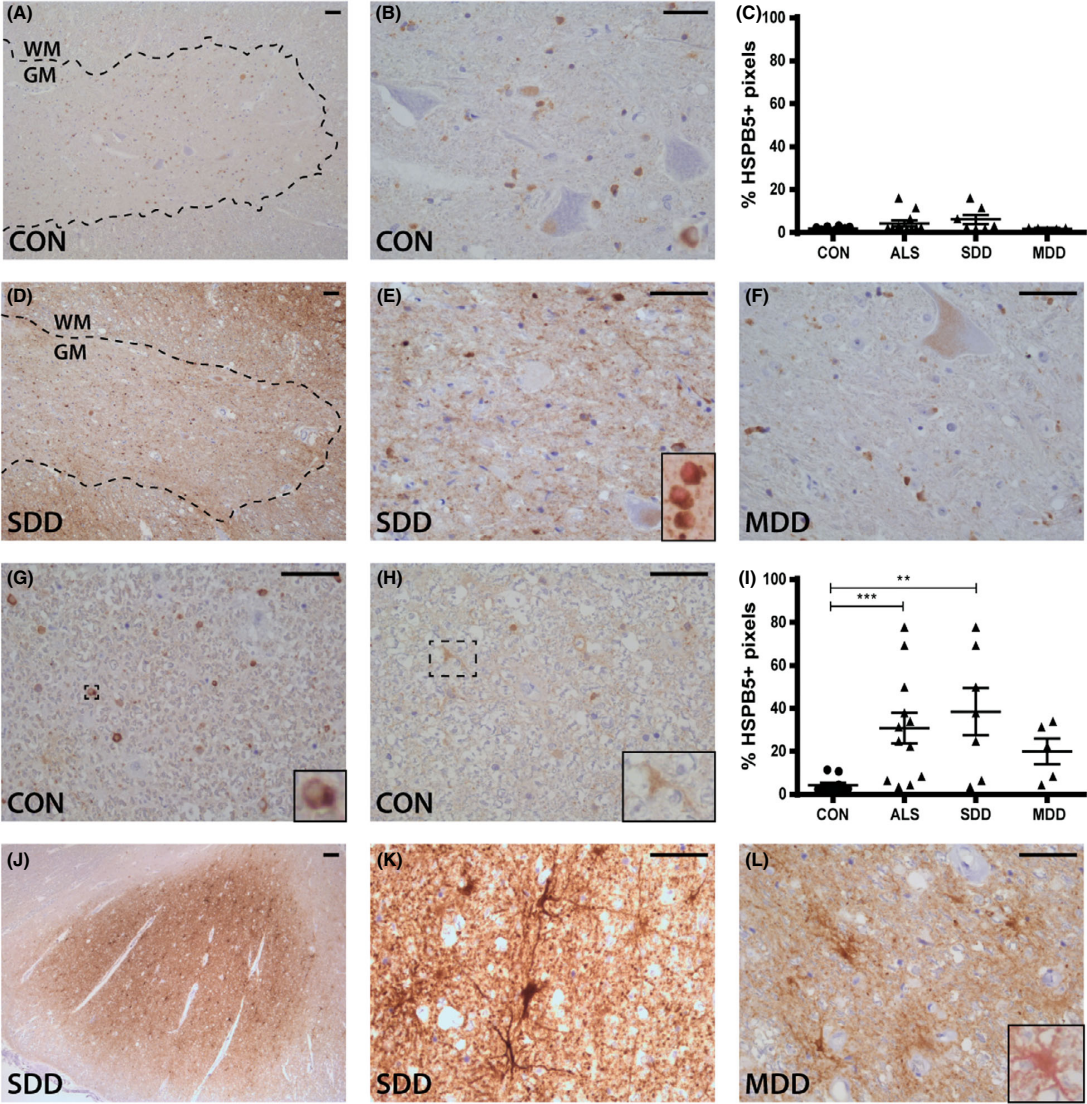
HSPB5 expression in ALS spinal cord. HSPB5 expression in (**A**,**B**,**D**–**F**) ventral horns and (**G**,**H**,**J**–**L**) lateral tracts of controls and ALS patients (subgroups: SDD and MDD) with inserts of (**E**) oligodendrocyte transcription factor 2 (olig2+) (pink) and HSPB5+ (brown) oligodendrocytes and (**L**) a vimentin+ (pink) and HSPB5+ (brown) astrocyte. Grey matter is delineated with a dotted line. Quantification of HSPB5+ pixels in (**C**) ventral horns and (**I**) lateral columns of controls and ALS patients (subgroups: SDD and MDD). Data points represent the mean value for each patient. Data are shown as mean ± SEM. Significance was analysed between ALS patients (*n* = 12) and controls (*n* = 10) with Student's *t‐*test or Mann–Whitney *U*‐test. ALS patients with SDD (*n* = 7) and MDD (*n* = 5) were compared to controls (*n* = 10) using anova and Tukey's post‐test or Kruskal–Wallis *H* test and Dunn's *post hoc* multiple comparisons test. Significant data are presented (****P = <0.0001, ***P = <0.001, **<0.01, *P = <0.05). Scale bar in all pictures = 50 μm. Inserts are digitally enlarged. SDD, short disease duration; MDD, moderate disease duration; ALS, amyotrophic lateral sclerosis; HSPB, heat shock protein B.

HSPB5, was not observed in the motor neurons in the ventral horns of controls (Figure 4 **A**–**C**) nor ALS cases (Figure 4 **C**–**F**). Rather, expression in the ventral horns was almost exclusively present in the cytoplasm of olig2+ cells (Figure 4 **E**). In three ALS SDD cases, HSPB5 was diffusely expressed in the ventral horn in regions close to the lateral tracts that contained HSPB5+ astrocytes, and from which the processes extended into the grey matter (Figure 4 **D**,**E**). In contrast to the ventral horns, HSPB5 was expressed in astrocyte‐like cells throughout the white matter (Figure 4 **G**) and around the central canal (Figure 4 **H**) in controls. In comparison, HSPB5 was highly expressed in ALS cases (*P* = 0.0006; Figure 4 **I**), especially in SDD cases (*P* = 0.0055; Figure 4 **I**,**J**,**K**). Such expression was localized to nuclei, cell bodies and astrocytes, as confirmed by double staining with vimentin (Figure 4 **L**, insert).

**Figure 5:**
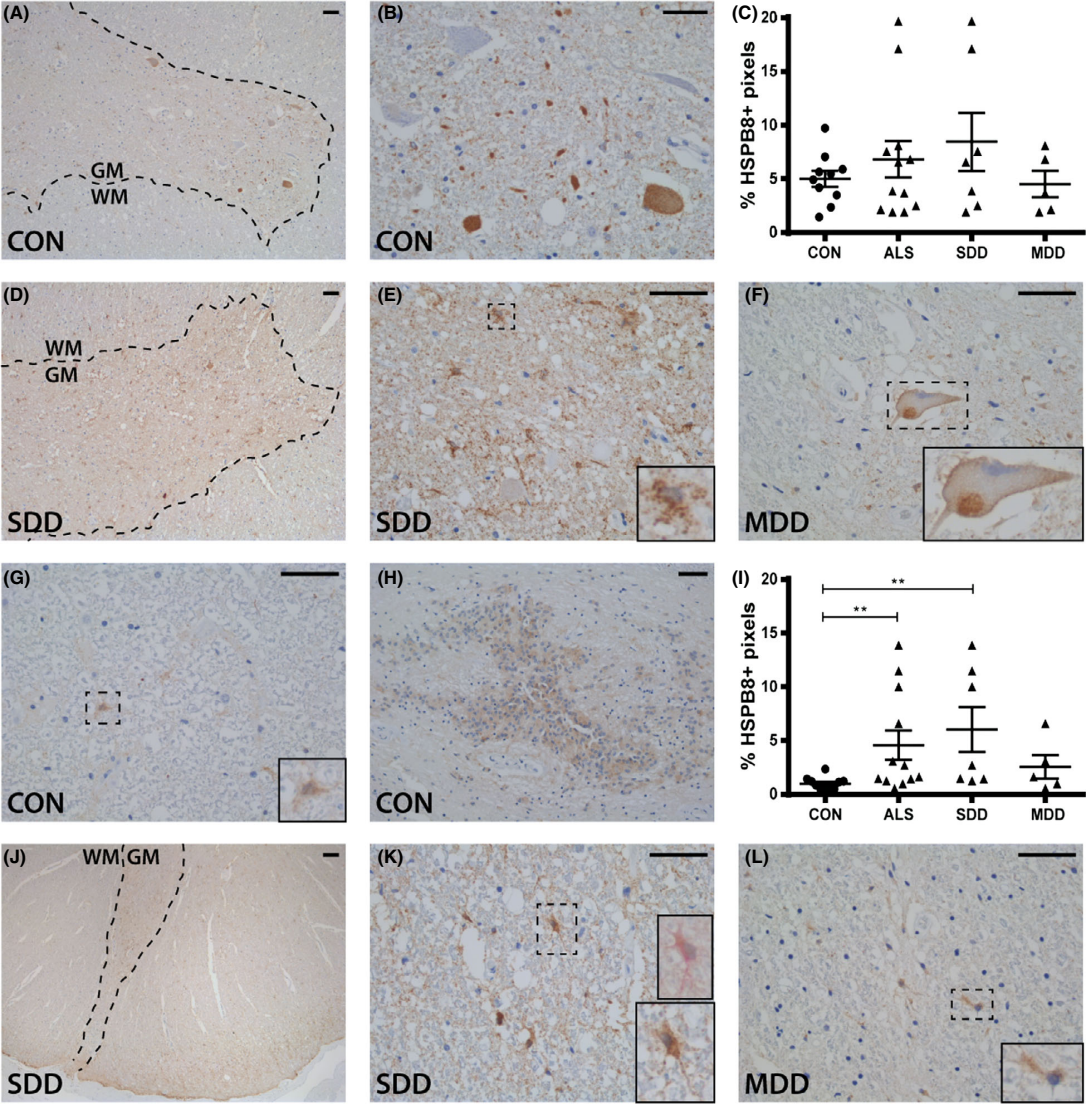
HSPB8 expression in ALS spinal cord. HSPB8 expression in (**A**,**B**,**D**–**F**) ventral horns and (**G**,**H**,**J**–**L**) lateral tracts of controls and ALS patients (subgroups: SDD and MDD) with (**K**) an insert of a vimentin+ (pink) and HSPB8+ (brown) astrocyte. Grey matter is delineated with a dotted line. Quantification of HSPB8+ pixels in (**C**) ventral horns and (**I**) lateral columns of controls and ALS patients (subgroups: SDD and MDD). Data points represent the mean value for each patient. Data are shown as mean ± SEM. Significance was analysed between ALS patients (*n* = 12) and controls (*n* = 10) with Student's *t*‐test or Mann–Whitney *U*‐test. ALS patients with SDD (*n* = 7) and MDD (*n* = 5) were compared to controls (*n* = 10) using anova and Tukey's post‐test or Kruskal–Wallis *H* test and Dunn's *post hoc* multiple comparisons test. Significant data are presented (****P = <0.0001, ***P = <0.001, **<0.01, *P = <0.05). Scale bar in all pictures = 50 μm. Inserts are digitally enlarged. SDD, short disease duration; MDD, moderate disease duration; ALS, amyotrophic lateral sclerosis; HSPB, heat shock protein B.

Different from HSPB5, HSPB8 was strongly expressed in cell bodies and axons of ventral horn neurons in both controls and ALS (Figure 5 **A**,**B**,**D**–**F**). Rarely, HSPB8+ inclusions were observed (Figure 5 **F**). HSPB8 was also expressed in occasional grey matter astrocytes in controls and in two ALS patients with SDD. Overall, ventral horn HSPB8 expression showed no difference between groups (*P* > 0.9999; Figure 5 **L**). In controls, HSPB8 expression was occasionally observed in nuclei of cells with an astrocyte‐like morphology (Figure 5 **G**) throughout the lateral tracts and the central canal (Figure 5 **H**). In ALS, the number of HSPB8+ vimentin+ astrocytes was higher in lateral tracts, and where expression extended into astrocyte cell bodies and processes (Figure 5 **J**–**L**).

### HSPB6 expression is constitutively expressed in the spinal cord

**Figure 6:**
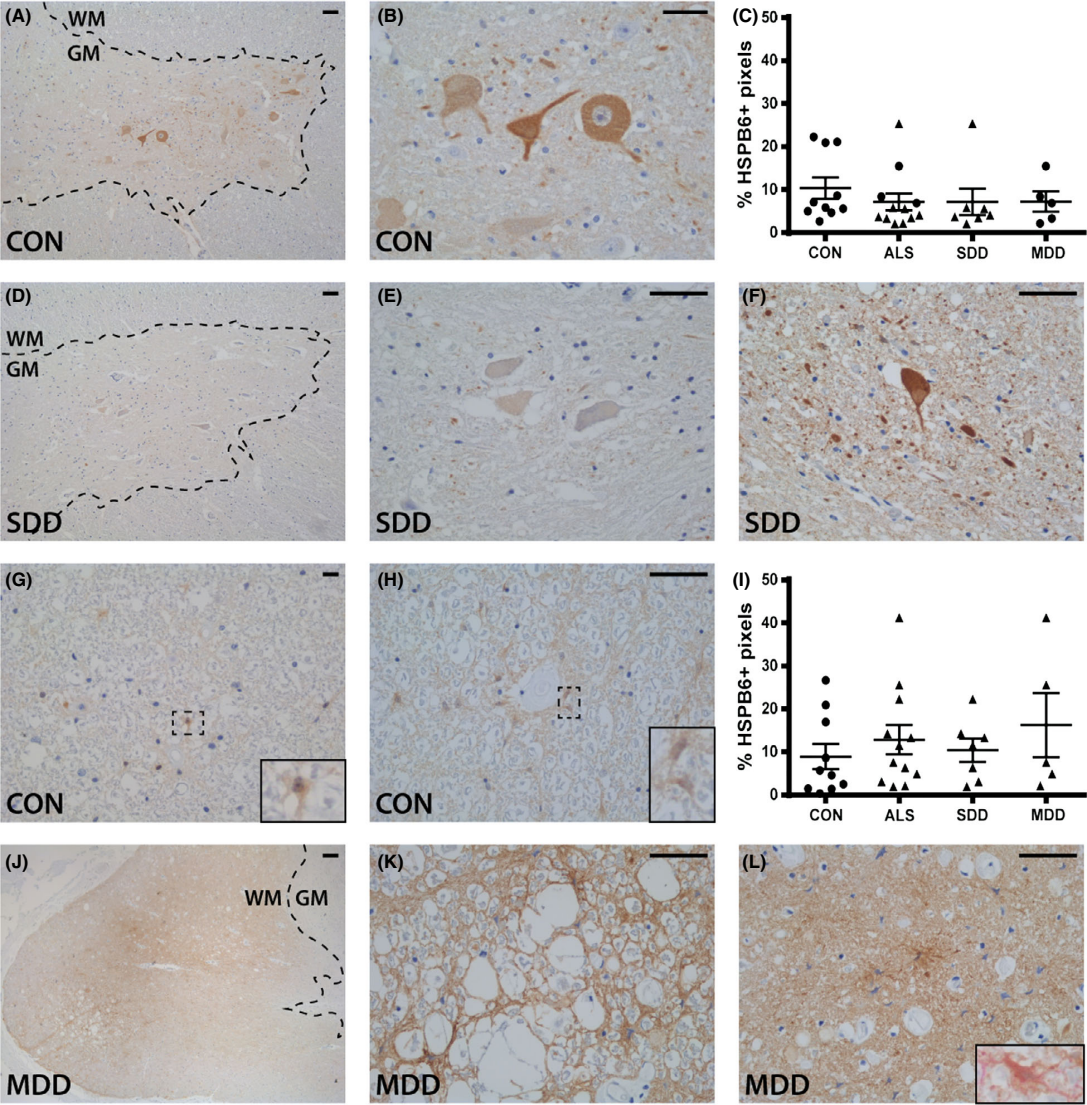
HSPB6 expression in ALS spinal cord. HSPB6 expression in (**A**,**B**,**D**–**F**) ventral horns and (**G**,**H**,**J**–**L**) lateral tracts of controls and ALS patients (subgroups: SDD and MDD) with (**L**) an insert of a vimentin+ (pink) and HSPB6+ (brown) astrocyte. Grey matter is delineated with a dotted line. Quantification of HSPB6+ pixels in (**C**) ventral horns and (**I**) lateral columns of controls and ALS patients (subgroups: SDD and MDD). Data points represent the mean value for each patient. Data are shown as mean ± SEM. Significance was analysed between ALS patients (*n* = 12) and controls (*n* = 10) with Student's *t*‐test or Mann–Whitney *U*‐test. ALS patients with SDD (*n* = 7) and MDD (*n* = 5) were compared to controls (*n* = 10) using anova and Tukey's post‐test or Kruskal–Wallis *H* test and Dunn's *post hoc* multiple comparisons test. Scale bar in all pictures = 50 μm. Inserts are digitally enlarged. SDD, short disease duration; MDD, moderate disease duration; ALS, amyotrophic lateral sclerosis; HSPB, heat shock protein B.

Low expression of HSPB6 was observed in neuronal cell bodies, axons and occasional grey matter astrocytes in control (Figure 6 **A**,**B**) and ALS cases (Figure 6 **D**–**F**), although no differences in HSPB6 expression were detected (Figure 6 **C**). HSPB6+ inclusions were not observed.

In the lateral tracts of control cases, HSPB6 expression was observed in cells resembling astrocytes (Figure 6 **G**), which was more pronounced adjacent to blood vessels (Figure 6 **H**). No differences in expression between ALS and control cases were detected (Figure 6 **I**); however, three ALS patients revealed relatively high expression of HSPB6 in the lateral tracts (Figure 6 **J**–**L**). Here, HSPB6 staining was both diffuse and localized to astrocytes in areas of tissue damage (Figure 6 **J**–**L**).

### HSP16.2 expression is increased in lateral columns in ALS with long disease duration

**Figure 7:**
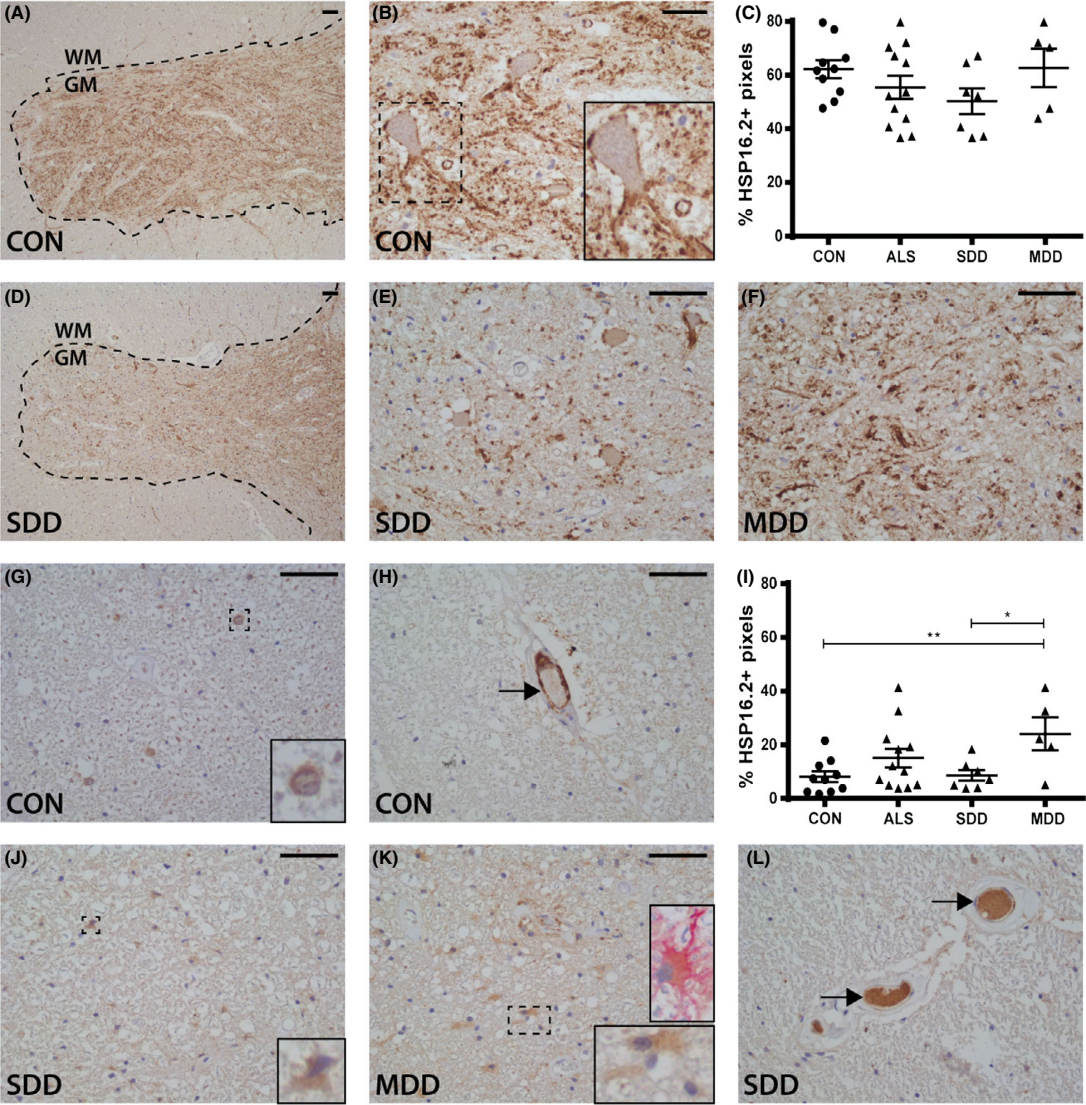
HSP16.2 expression in ALS spinal cord. HSP16.2 expression in (**A**,**B**,**D**–**F**) ventral horns and (**G**,**H**,**J**–**L**) lateral tracts of controls and ALS patients (subgroups: SDD and MDD) with (**K**) an insert of a vimentin+ (pink) and HSP16.2+ (brown) astrocyte. HSP16.2+ blood vessels are indicated with arrows (**H**,**L**). Grey matter is delineated with a dotted line. Quantification of HSP16.2+ pixels in (**C**) ventral horns and (**I**) lateral columns of controls and ALS patients (subgroups: SDD and MDD). Data points represent the mean value for each patient. Data are shown as mean ± SEM. Significance was analysed between ALS patients (*n* = 12) and controls (*n* = 10) with Student's *t*‐test or Mann–Whitney *U*‐test. ALS patients with SDD (*n* = 7) and MDD (*n* = 5) were compared to controls (*n* = 10) using anova and Tukey's post‐test or Kruskal–Wallis *H* test and Dunn's *post hoc* multiple comparisons test. Significant data are presented (****P = <0.0001, ***P = <0.001, **<0.01, *P = <0.05). Scale bar in all pictures = 50 μm. Inserts are digitally enlarged. SDD, short disease duration; MDD, moderate disease duration; ALS, amyotrophic lateral sclerosis; HSP heat shock protein.

Extensive HSP16.2 expression was observed on the cell membrane of neurons and axons throughout the grey matter of controls (Figure 7 **A**–**C**). Similar expression was observed in ALS cases (Figure 7 **D**–**F**) in the grey matter but not in the ventral horns where staining was less intense (non‐significant; Figure 7 **C**). The numbers of sharply delineated HSP16.2+ motor neurons were decreased (Figure 7 **E**) and expression associated with tissues with a fibrotic appearance (Figure 7 **F**). No HSP16.2+ inclusions were observed.

In controls, HSP16.2 expression was observed occasionally in nuclei of astrocyte‐like cells throughout the lateral tracts (Figure 7 **G**), surrounding the central canal (data not shown) and occasionally in endothelial cells (Figure 7 **H**). HSP16.2 expression was significantly higher in MDD cases compared to controls (*P* = 0.0059; Figure 7 **I**,**K**) and SDD cases (*P* = 0.9931; Figure 7 **J**). In some ALS cases and controls, HSP16.2 was also observed in the lumen of blood vessels (Figure 7 **L**).

## Discussion

Although motor neurons degenerate in ALS, the exact pathological mechanisms underlying functional decline and disease progression are unknown 27. Here, we investigated the association between ALS disease duration, motor neuron damage and expression of the stress‐inducible HSPBs as markers of glial activation and inflammation in ALS spinal cord. We show that although the motor neurons in ALS spinal cord do not upregulate basal expression of HSPB1, 6, 8 and HSP16.2, expression of several HSPBs is markedly increased in astrocytes in the lateral columns throughout the spinal cord. This suggests these cells play a role in the pathogenesis of ALS. Moreover, we show that SDD is associated with less motor neuron loss, more microglial activity and increased HSPB5 and 8 expression. This possibly indicates a relationship in ALS between stressors, such as increased proteostasis and oxidative stress and astrocyte reactivity, microglial activation and survival.

HSPBs, including HSPB1, 5 and 8 may aid motor neuron survival by preventing protein aggregation 18, 19, 20. Although HSPB1 and 8 mRNA levels have previously been found to be increased in ALS ventral horns 28, our study shows that only very few neurons contained HSPB1+ or HSPB8+ inclusions. Moreover, we did not observe increased expression of HSPBs in ventral horns during ALS, but rather a decrease in expression of HSPB1. This could be due to loss of motor neurons or decreased expression by residual motor neurons, similar to decreased HSPB1 protein expression in SOD1^G93A^ mice, which precedes motor neuron degeneration 29. Generally, the lack of HSPB upregulation in ALS motor neurons is consistent with the concept that motor neurons have a high threshold before upregulating HSPs in response to heat shock or protein inclusions 30, 31.

In contrast to expression of HSPB in the motor neurons, a marked expression of HSPB5, 8 and HSP16.2 was observed in astrocytes in the lateral tracts. HSPB upregulation in astrocytes is prominent in many neurological diseases, for example multiple sclerosis, Alzheimer's disease, Parkinson's disease and X‐linked adrenoleucodystrophy and is associated with astrocyte reactivity 11, 13, 14, 15. In ALS, upregulation of HSPBs in astrocytes in response to for example oxidative stress, inflammation, neurotoxic protein aggregation or neuronal damage may thus confer protection by exerting anti‐apoptotic and anti‐protein aggregating effects 32. Simultaneously, HSPBs might facilitate astrocyte activation as their molecular chaperoning functions are important in cytoskeletal maintenance and reorganization 33. HSPB5 associates with astrocytic cytoskeletal proteins such as GFAP 34, and expression and phosphorylation of HSPB5 have recently been implicated in astrogliosis 35, which is prominent in ALS 36, 37. The increase in HSPB was not due to an increase in astrocyte numbers *per se* as GFAP and ALDH1 levels did not differ from controls. Astrocytes in ALS contain inclusions and exhibit an altered phenotype, likely contributing to motor neuron death 8, 38, 39, 40, 41, 42. As astrogliosis may be a physiological response necessary for tissue repair as well as playing a pathogenic role, the actual role that HSPBs play in astrocytes during ALS remains to be determined. Of note, not all HSPBs are consistently upregulated in ALS spinal cord (that is HSPB1 and 6), while HSPB5 and 8 are upregulated in patients with SDD, HSP16.2 is increased in ALS cases with MDD, underscoring the functional diversity of the different HSPB family members 16.

In our study, SDD is associated with increased HSPB5 and 8 expression in astrocytes, increased activation of microglia and/or macrophages, and relatively little motor neuron loss. That SDD cases have similar levels of pTDP‐43 pathology compared with MDD patients, implying that neuronal pathology alone does not explain disease progression in ALS. The limited motor neuron pathology in SDD ALS could indicate that in these particular cases, death was due to neuromuscular junction pathology, Wallerian degeneration or respiratory failure, prior to extensive motor neuron cell body loss in the spinal cord. In serum of ALS patients, HSP70 and HSP90 levels are reported to be high in early disease, but gradually decline with disease progression 43. Moreover, in animal models and human cell lines, prolonged stress depletes cellular levels of HSPs leading to maladaptation of the protein quality control system 32, 44. Thus, reduced HSPB expression and microglial activation in patients with MDD may reflect an exhausted immune and stress response resulting in a transition from acute to chronic inflammation. Further studies are needed to further characterize the inflammatory profile of glial cells in ALS with SDD and MDD.

Examination of *post mortem* tissue limits the separation of causative factors from reactive responses. For example, the heightened inflammatory response in patients with SDD may be a feature of early ALS, or alternatively a causative factor in disease progression. As SDD is associated with relative preservation of neurons and only moderate levels of pTDP‐43 inclusions, spinal cord neuronal pathology itself does not explain the rapid clinical decline of these patients. In ALS brain, the density of TDP‐43 inclusions does not correlate with disease duration or with rate of progression 45. Rather, glial responses to motor neuron degeneration may determine disease severity. Indeed, several studies in humans seeking factors contributing to disease progression have found an increased presence of pro‐inflammatory markers in CSF 46 and blood 47, 48 of patients with rapidly progressive ALS. Moreover, a recent data‐driven approach evaluating the ALS transcriptome, neuropathology and genome wide associations, underscored the link between microglia activation and disease progression in ALS 49. Furthermore, reduction of microglial proliferation in *SOD1* ^*G93A*^ mice is associated with less motor neuron death, reduced disease progression and prolonged survival 50. Nevertheless, a recent study established that in a TDP‐43^*rNLS8*^ mouse model of ALS the microglial response is subtle and neuroprotective 51. The same study observed robust microglial activity in patients with SOD1 mutations in contrast to variable microglial reactivity in sporadic ALS (sALS) patients. The authors hypothesized that this observation is due to the diverse nature of the toxic aggregates in sALS patients, which may be due to unidentified mutations and/or environmental factors. Given the current lack of knowledge regarding the nature of the aggregates in sALS cases it is difficult to test this hypothesis. Our studies indicate that rather than the nature of the aggregates, the level of microglial activation in sALS patients is strongly associated with disease duration.

## Conclusions

Our findings show that HSPBs are upregulated predominantly in astrocytes in ALS. These findings support the hypothesis that the interaction between motor neurons, microglia and astrocytes determines neuronal fate and thus functional decline in ALS. Modulating the pathogenic response and harnessing the protective response of innate immunity in the CNS may favourably impact the disease course in ALS.

## Author contributions

RPG, JS, EN, JCJ and M‐CJ performed the experiments. JA, CM and EA provided clinical material and data regarding patient characteristics. JB and WB provided technical support for the analysis. RPG, JS, EN, HvN and SA wrote the paper. All authors contributed and agreed to the final version of the paper.

## Supporting information


**Table S1.** Antibodies for IHC.

**Appendix S1.** Macro used for determining DAB+ area.
