# Supplementary material for: Rapidly progressive amyotrophic lateral sclerosis is associated with microglial reactivity and small heat shock protein expression in reactive astrocytes
Source: Neuropathol Appl Neurobiol. 2018 Nov 23;45(5):459–75. doi: 10.1111/nan.12525 (PMC7379307; doi:10.1111/nan.12525)
Supplement: Supplementary file 1 — Table S1. Antibodies for IHC. [file NAN-45-459-s001.docx]

**SUPPLEMENTARY INFORMATION**

*Supplementary table 1: Antibodies for IHC*

| A) Primary antibodies | | | | | | | | |  |
| --- | --- | --- | --- | --- | --- | --- | --- | --- | --- |
| **Antigen** | **Antibody number** | **Species** | **Clone** | **Dilution IHC** | | **Antigen retrieval** | **Incubation time** | | **Company** |
| pTDP-43 | CAC-TIP-PTD-M01 | mouse | monoclonal | 1:8000 | | citrate buffer | 1 hour | | Cosmobio |
| HLA-DR | 14-99-56-82 | mouse | monoclonal | 1:1000 | | citrate buffer | 1 hour | | eBioscience |
| HSPB1 | AB155987 | rabbit | monoclonal | 1:1500 | | citrate buffer | O/N | | Abcam |
| HSPB5 | JAM01 | mouse | monoclonal | 1:750 | | TRIS/EDTA | 1 hour | | in house |
| HSPB6 | AB184161 | rabbit | monoclonal | 1:50000 | | citrate buffer | O/N | | Abcam |
| HSPB8 | AB96837 | rabbit | polyclonal | 1:3500 | | citrate buffer | O/N | | Abcam |
| HSP16.2 | NBP1-88332 | rabbit | polyclonal | 1:50 | | citrate buffer | O/N | | Novus |
| olig2 | AB9610 | rabbit | polyclonal | 1:750 | | citrate buffer | overnight | | Millipore |
| GFAP | AB5541 | chicken | polyclonal | 1:1000 | | citrate buffer | overnight | | Chemicon International |
| ALDH1 | AB52492 | rabbit | monoclonal | 1:500 | | citrate buffer | overnight | | Abcam |
| vimentin | V9 | rabbit | mouse | 1:128000 | | citrate buffer | 1 hour | | in house |
| B) Secondary antibodies | | | | | | | | | |
| **Antigen** | | | **Species** | | **Dilution** | | | **Company** | |
| EnVision HRP anti rabbit | | | goat | | undiluted | | | Dako | |
| EnVision HRP anti mouse | | | goat | | undiluted | | | Dako | |
| Goat-anti-Mouse AP | | | goat | | 1:250 | | | Dako | |
| Goat-anti-rabbit AP | | | goat | | 1:250 | | | Southern Biotech | |

*ALDH1 – aldehyde dehydrogenase 1; AP – alkaline phosphatase; GFAP – glial fibrillary acid protein; HLA-DR – human leukocyte antigen DR; HRP – horseradish peroxidase; HSP – heat shock protein****;*** *HSPB – small heat shock protein; olig2 – oligodendrocyte transcription factor 2*
