# Supplementary material for: Rapidly progressive amyotrophic lateral sclerosis is associated with microglial reactivity and small heat shock protein expression in reactive astrocytes
Source: Neuropathol Appl Neurobiol. 2018 Nov 23;45(5):459–75. doi: 10.1111/nan.12525 (PMC7379307; doi:10.1111/nan.12525)
Supplement: Supplementary file 2 — Appendix S1. Macro used for determining DAB+ area. [file NAN-45-459-s002.docx]

*Supplementary information 2: Macro used for determining DAB+ area*

path=getDirectory()

list=getFileList(path);

for(i=0;i<list.length;i++){

open(path+list[i]);

imageTitle=getTitle();

run("Colour Deconvolution", "vectors=[User values] [r1]=0.38523284 [g1]=0.6003067 [b1]=0.7008763 [r2]=0.63229185 [g2]=0.6532448 [b2]=0.41650718 [r3]=0.5753916 [g3]=0.5760428 [b3]=0.5806247");

selectWindow(imageTitle+ "-(Colour_1)");

//run("Threshold...");

setThreshold(0,X);

run("Measure");

}
